# Unraveling Facial Nerve Palsy: A Case Series Highlighting Diagnostic and Therapeutic Challenges

**DOI:** 10.7759/cureus.71445

**Published:** 2024-10-14

**Authors:** Deepankumar T, Madhumitha Selvaraju, Krishnaswamy Madhavan, Janardanan Kumar

**Affiliations:** 1 Department of General Medicine, Sri Ramaswamy Memorial (SRM) Medical College Hospital and Research Centre, Sri Ramaswamy Memorial (SRM) Institute of Science and Technology, Chengalpattu, IND

**Keywords:** arachnoid cyst, lyme disease, seventh cranial nerve palsy, unilateral facial nerve palsy, vascular loop compression

## Abstract

Facial nerve palsy (FNP) may arise from multiple etiological reasons, including anatomical anomalies, circulatory complications, and infectious agents. This case series underscores the importance of a comprehensive diagnostic approach to identify the precise etiology, including structural abnormalities, vascular anomalies, or infectious illnesses. Here, we present three distinct occurrences of FNP, emphasizing the varied diagnostic difficulties and therapeutic strategies. It includes an arachnoid cyst, which when occurring at the cerebellopontine angle can affect multiple cranial nerves, specifically the seventh and eighth cranial nerve, a neurovascular compression syndrome, where the anterior inferior cerebellar artery loops around the facial nerve. Most often, this scenario causes hemifacial spasms, which in our scenario manifests as FNP. Lyme disease is a tick-borne disease that affects multiple cranial nerves, specifically the facial nerve. Effective management necessitates a focused treatment strategy that tackles the symptoms and the underlying disease. Advanced imaging techniques, serological tests, and a tailored treatment approach are essential for effective diagnosis, can have significant implications for patient well-being, and necessitate a thorough evaluation to identify underlying causes. This case series illustrates the diverse etiologies of FNP, emphasizing the need for comprehensive diagnostic strategies and targeted treatments. As clinicians encounter FNPs more often, this case series can help physicians understand facial palsy better. Continuous research and clinical awareness are vital for improving patient outcomes in cases of FNP.

## Introduction

The facial nerve is an essential structure for both emotion and communication; its functional damage can significantly lower the quality of life. Bell’s palsy (BP), also known as lower motor neuron (LMN) facial nerve palsy (FNP), has an enigmatic etiology and a frequency of 10-40 per 100,000 people [1]. This condition typically manifests as complete unilateral palsy and LMN damage. Despite being believed to be a viral prodromal in most cases, the symptoms of FNP often become completely apparent within the first 24 to 48 hours, with a recurrence occurring in 10% of cases [2]. FNP can affect people of all ages, and there is currently no conclusive evidence suggesting that any gender or race is more likely to experience it. People with FNP typically fall within the 15-45-year age range, as widely recognized [3]. Although most cases remain unexplained, it is important to rule out a cerebral vascular accident and other underlying conditions. Bilateral FNP occurs in 0.3%-2% of all cases [4]. Overall, 35% of patients with Lyme disease (LD) have bilateral FNPs, making LD a prominent factor. Additional notable illnesses to consider include multiple sclerosis, sarcoidosis, Guillain-Barré syndrome, Parkinson’s disease, and bulbar palsy, which may cause bilateral FNP [4]. FNP is also rarely encountered if one has a membrane or vessel defect such as an arachnoid cyst (AC) or intracranial vessel loop. The purpose of this study is to systematically examine three distinct cases of FNP to highlight the complexities in diagnosis and management. By doing so, we aim to identify key factors influencing treatment outcomes and enhance the understanding of effective management practices in clinical settings.

## Case presentation

Case 1

**Figure 1:**
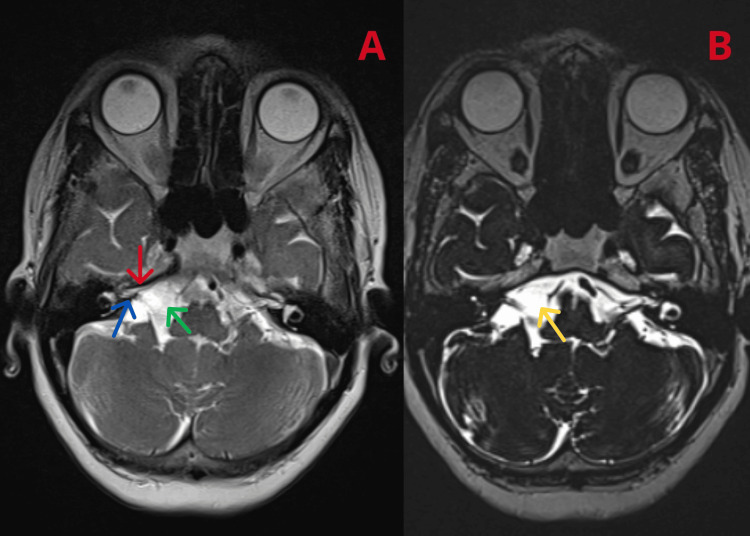
MRI of the brain with MRA and MRV showing (A) CSF signal intensity area in the right CP angle (~2.1 × 1.2 × 0.9 cm) causing a widening of the lateral cerebellar medullary cistern abutting the seventh (red arrow) and eighth (blue arrow) cranial nerves, its inferior aspect, and AICA branches diagnostic of arachnoid cyst (green arrow in A and yellow arrow in B). MRI: magnetic resonance imaging; MRA: magnetic resonance angiography; MRV: magnetic resonance venography; AICA: anterior inferior cerebellar artery; CSF: cerebrospinal fluid; CP: cerebellopontine

A 65-year-old female, known to be diabetic, hypertensive, and hypothyroid, presented with a right-sided accumulation of food in the mouth and an inability to close the right eye with occasional giddiness, progressive over one year. The patient was treated with native medication. Her symptoms worsened over the past two months, and she developed a mild sensory neural hearing loss (SNHL) on the right side two months ago. Examination revealed the patient’s inability to wrinkle her right forehead, inability to fully close her right eye, and a deviated mouth angle to the left, all of which were indicative of right-sided FNP. The eighth nerve examination Rinne test was normal. Weber test lateralized to the left ear indicating SNHL in the right ear. The fifth nerve examination revealed impaired corneal reflex. No limb weakness was elicited, and cerebellum examination was normal. The external ear was normal, and there was no mastoid tenderness. Magnetic resonance imaging (MRI) of the brain with magnetic resonance angiography (MRA) and magnetic resonance venography (MRV) (Figure 1) showed cerebrospinal fluid (CSF) signal intensity area in the right cerebellopontine (CP) angle (~2.1 × 1.2 × 0.9 cm) causing widening of the lateral cerebellar medullary cistern abutting the seventh and eighth cranial nerves, its inferior aspect, and anterior inferior cerebellar artery (AICA) branches.

A diagnosis of AC in the CP angle was made. A neurologist’s opinion was sought who advised initiating steroids with facial nerve stimulation. The neurosurgery opinion recommended no surgical intervention, suggested a conservative approach, and requested a review if the patient’s symptoms did not improve with medical management. The patient’s symptoms improved significantly with facial nerve stimulation and steroid therapy.

Case 2

**Figure 2:**
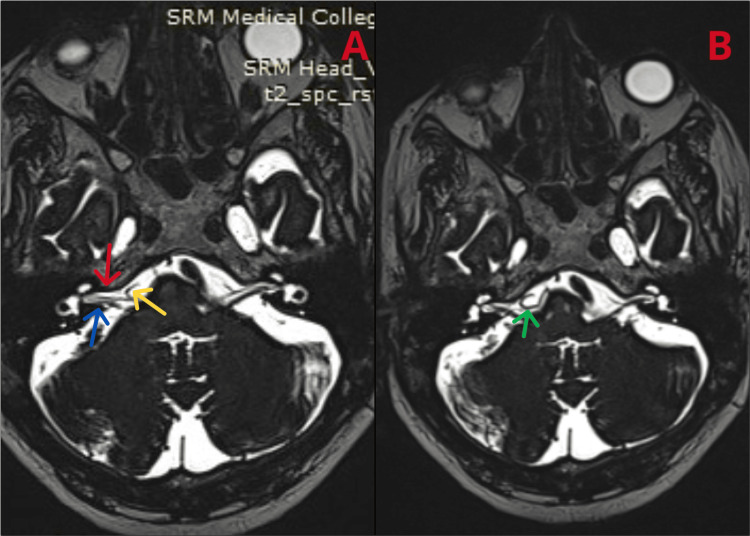
MRI of the brain revealing a (A) vascular loop (yellow arrow) of the AICA abutting the seventh (red arrow) and eighth (blue arrow) cranial nerve complexes on the right side in the CP angle cistern and (B) the AICA loop (green arrow). MRI: magnetic resonance imaging; MRA: magnetic resonance angiography; MRV: magnetic resonance venography; AICA: anterior inferior cerebellar artery; CP: cerebellopontine

A 60-year-old male with diabetes and hypertension on regular medications presented to an outside hospital with complaints of drooling saliva on the right side of the mouth and difficulty closing the right eye, with the symptoms being sudden in onset. The patient’s examination revealed a deviated left mouth angle and an inability to fully close the right eye, despite the external ear appearing normal. At the outside hospital, the patient was treated for idiopathic BP. The patient improved symptomatically after a month. He came to our outpatient department two months later with FNP on the same side. Given the recurrent FNP, we performed an MRI of the brain with MRA and MRV, revealing a radiological finding of a vascular loop of the AICA abutting the seventh and eighth cranial nerve complexes on the right side in the CP angle cistern (Figure 2), explaining the right FNP.

The patient did not report any symptoms of tinnitus or hearing loss related to the compression of the eighth nerve. A neurosurgery opinion was obtained, advising surgical intervention if the patient’s current episode did not resolve with conservative management. An ophthalmology opinion was also obtained because of FNP, suggesting starting steroids and lubricants. We initially started the patient on oral prednisolone at 40 mg/day, with plans to taper the steroids at the next review. The patient received facial nerve stimulation and lid tapping. At the next review, after seven days of treatment with steroids and without any surgical intervention, the patient improved symptomatically, with some residual weakness. Following the second episode, the patient recovered three months later with residual orbicularis oculi weakness.

Case 3

**Figure 3:**
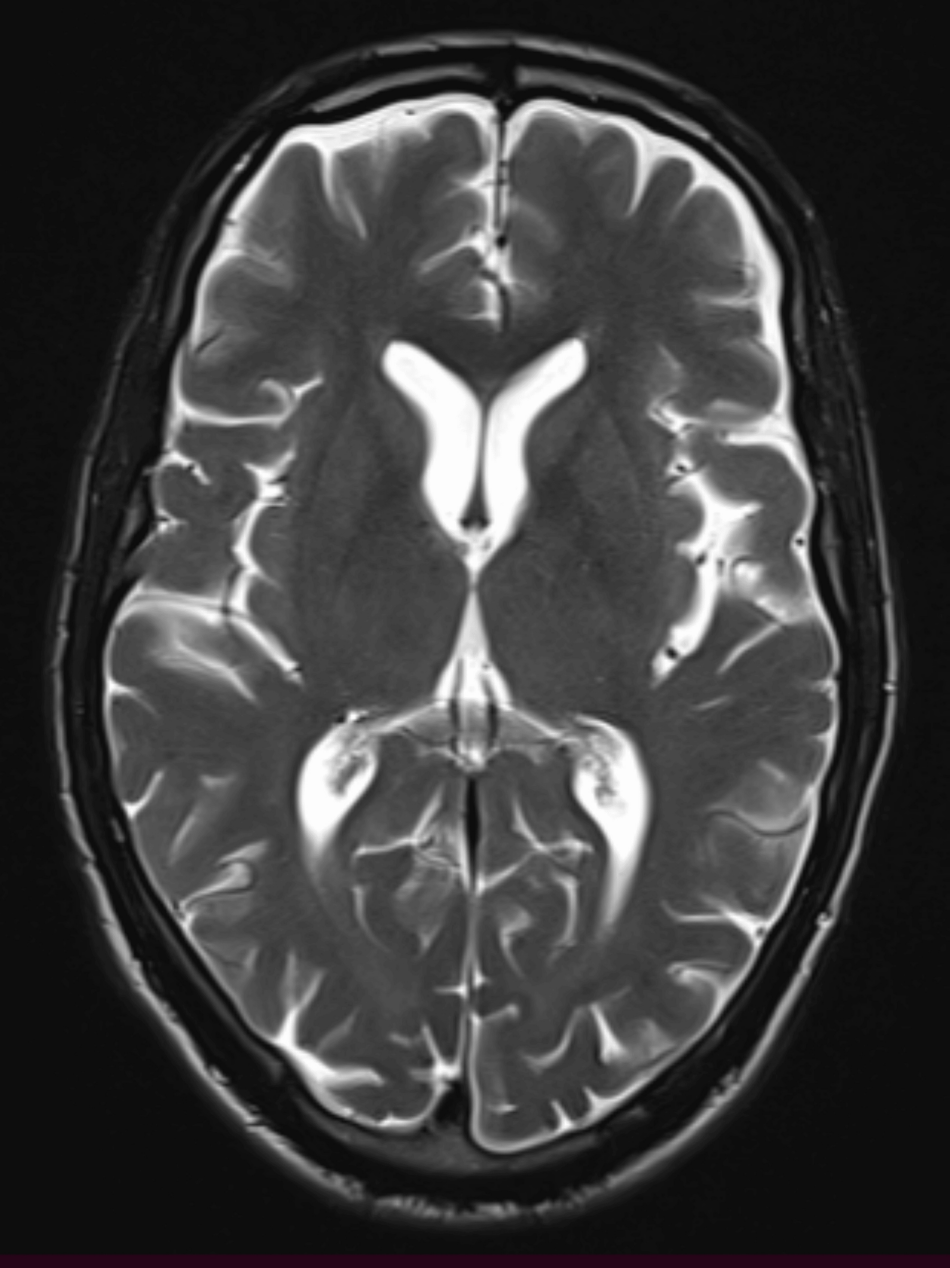
Contrast-enhanced T2-weighted MRI of the brain showing normal findings. MRI: magnetic resonance imaging

A 72-year-old male presented to the emergency room with complaints of difficulty swallowing for the past week. He also had a history of intermittent fever over the past three weeks. The patient had a left-facing uvula, a nasal twang in his voice, and no gag reflex. All other cranial nerves were normal. There was no elicitation of limb weakness, and plantar reflexes were bilaterally flexor. Given the patient’s difficulty swallowing, we performed an upper gastrointestinal endoscopy to rule out mechanical causes of dysphagia. With the assistance of a gastroenterologist, we inserted a percutaneous endoscopic gastrostomy (PEG) tube for feeding. During his hospital stay, the patient developed bilateral FNP. As multiple cranial nerves (seven, nine, and ten) were involved and he had a fever with a headache, we decided to perform CSF analysis and measure serum angiotensin-converting enzyme (ACE) levels (for sarcoidosis). The CSF analysis was normal, serum electrophoresis did not reveal anything significant, and ACE levels were within the normal range. A contrast-enhanced MRI of the brain was also normal (Figure 3).

To rule out infectious etiology, serology forEpstein-Barr virus and cytomegalovirusdisease came back negative. For LD, immunoglobulin M (IgM) enzyme-linked immunosorbent assay (ELISA), and western blot were performed. The results are summarized in Table 1. We conducted a Venereal Disease Research Laboratory test because syphilis can lead to false positive results for LD, but the results came back negative. Hence, a diagnosis of LD was made.

**Table 1:** Laboratory investigations. Ig: immunoglobulin; ELISA: enzyme-linked immunosorbent assay; OspA: outer surface protein A; OspB: outer surface protein B; BmpA: *Borrelia burgdorferi* basic membrane protein A

Test	Result	Biological reference
Western blot band (kDa)		
31 (OspA)	Positive	Negative
34 (OspB)	Positive	Negative
39 (BmpA protein)	Positive	Negative
41 (flagellin B protein)	Positive	Negative
Borrelia IgM (ELISA)	1.7 index	<0.9 = negative >1.1 = positive

The patient was started on intravenous ceftriaxone (2 g) once daily for 28 days. During the treatment, he showed symptomatic improvement, but residual weakness persisted. The patient improved symptomatically upon completion of the antibiotic course. The PEG tube was removed, he was able to close his eyes and wrinkle his eyebrows, and there was no deviation in the angle of the mouth.

## Discussion

Case 1

AC typically develops in the anterior, middle, or posterior cranial fossa. The male-to-female ratio is 2:1 [5]. For adults, the incidence ranges from 0.3% to 1.7% [5]. While the exact reason for most AC cases remains unclear, the formation of these cysts is most likely due to abnormal splitting that occurs during arachnoid embryogenesis [5]. One possible explanation for an AC is that CSF flow is uneven between the embryonic meninges. Typically, the pulse pressure of the CSF rises, causing a division between the brain coverings that form the subarachnoid space, pia, and arachnoid. When the layers of tissue seal in an unnatural way, CSF becomes trapped, resulting in the formation of an AC [6]. ACs are often incidentally detected in patients undergoing imaging studies for various reasons, such as headaches, stroke, cranial nerve paralysis, seizures, and tinnitus [5]. AC can cause a variety of symptoms, including diplopia, SNHL, hemifacial spasm, and cranial nerve dysfunction [7]. If the location of the AC is at the third ventricle along with cerebellar involvement, it can cause bobble-head doll syndrome [8]. Non-contrast MRI studies, such as three-dimensional (3D) T2-weighted sampling perfection with application-optimized contrast and varied flip-angle evolutions, constructive interference in steady-state, and rapid imaging utilizing steady-state acquisition, are among the methods that can be used. The results of these studies can help us understand how the AC connects to the surrounding structures [9]. The treatment of choice is surgery. Surgically removing the wall of the lesion while simultaneously creating a link with the subarachnoid region is performed when the lesion is readily amendable [10]. To open the AC cavity into the most adjacent portion of the ventricular system, we use either surgical fenestration or endoscopic fenestration [8]. Although surgery is the treatment of choice, recurrences of the AC have been documented [11].

Case 2

Vascular compression of the seventh nerve, especially at the CP angle, can cause FNP. The seventh nerve does not have any connective tissue septa between the nerve fascicles at the CP angle which is also where the central myelin, produced by oligodendrocytes, meets the peripheral myelin, which is produced by Schwann cells. These two distinctive features make the nerve particularly vulnerable to triggers such as compression or irritation [12]. Most neurovascular cross-compression causes hemifacial spasms, but seventh nerve palsy is also reported in some cases [13]. Once a clinical diagnosis of FNP is made, any vascular anomalies should be identified using an MRI of the brain. In particular, T2-weighted imaging and MRI angiography are employed [14]. After diagnosis, we can treat the paralysis with physiotherapy [13]. However, surgery is the treatment of choice, and microvascular decompression is the preferred procedure.

Case 3

The *Borrelia burgdorferi* bacterium causes LD and is transmitted through the bite of an *Ixodes* tick. Despite the typical prevalence of the disease in temperate regions, reports of *Ixodes* ticks carrying it, such as *Ixodes kashmiricus*, *I. granulatus*, *I. acutitarsus*, *I. himalayensis*, and *I. ovatus*, in the Himalayan region, suggest its potential presence in our country. A study published in the medical journal Armed Forces India in January 2008 showed that the prevalence of LD is 13% with more cases found in Arunachal Pradesh [15]. LD has three stages of clinical presentation, but one stage can overlap with another at any point in the course of the illness. Stage 1 lasts for 30 days and presents with local manifestations such as erythema migrans and flu-like symptoms. Stage 2 lasts for 3-10 weeks, which is otherwise called an early disseminated disease causing myalgia, fever, and fatigue, involves the musculoskeletal system, and may or may not involve the neurological system, cardiac (atrioventricular block, arrhythmia, carditis) system, and, rarely, the eye. Stage 3 spans from months to years and is primarily characterized by Lyme arthritis, nerve palsy, and the involvement of muscles. Neurological manifestations include lymphocytic meningitis, cranial nerve palsies, cerebellar ataxia (rarely), peripheral neuropathy, radiculopathy (Bannwarth syndrome), mononeuropathy multiplex, and encephalomyelitis (infrequently). LD has non-specific clinical features, and the vector, which is a tick, can transmit other infections such as *Babesia microti* and *Ehrlichia*. Overall, a prevalence of 10% of coinfection has been reported [16]. Meningeal seeding of the causative organism leads to central nervous system (CNS) manifestations as early as stage 2. The classic triad of early CNS involvement is (1) meningitis, (2) seventh nerve involvement, and (3) radiculoneuritis. Meningitis and FNP occur most of the time. Other cranial nerves (third, eighth, and fifth) can also be involved, including the lower cranial nerves [17]. In children, LD and FNP occur in 50% of cases, but in adults, it decreases to 10% [18]. The imaging investigation of choice is contrast-enhanced MRI [19]. Early LD is a clinical diagnosis characterized by erythema migrans. Researchers rarely use reverse transcriptase-polymerase chain reaction and culture with skin biopsy due to their poor sensitivity [19]. The Centers for Disease Control and Prevention recommends a two-step process for LD diagnosis. In the first step, IgM and IgG ELISA or immunoassay are performed. If the test results are positive or equivocal. the diagnosis is confirmed with a western blot for IgM and IgG, which is the second step [19]. The recommended treatment entails the use of an oral antibiotic, specifically doxycycline 100 mg twice daily, along with other oral agents such as amoxicillin and cefuroxime, all administered for 14 days. The preferred intravenous drug is ceftriaxone (2 g), administered intravenously for 28 days.

## Conclusions

Not all cases of FNP are benign. If they exhibit systemic symptoms, additional evaluation is necessary before labeling them as idiopathic. These cases highlight the need for a comprehensive diagnostic workup to determine the underlying causes of FNP and tailor treatment accordingly. The most effective way to improve patient outcomes and manage symptoms is to address the specific etiology, whether it be a structural anomaly, vascular issue, or infectious agent. It is essential to maintain ongoing research and awareness to enhance our understanding and treatment of these conditions.
